# Systematic Development and Validation of a Bradford-Based Protein Quantification Method for Novel Multi-Dose R21 Malaria Vaccine Formulated with 2-Phenoxy Ethanol (2-PE)

**DOI:** 10.3390/vaccines14010025

**Published:** 2025-12-24

**Authors:** Rajender Jena, Dnyanesh Ranade, Prajwal Chaudhari, Ajay Salunke, Aniket Mahamuni, Sunil Gairola

**Affiliations:** Quality Control Department, Serum Institute of India Pvt. Ltd., Hadapsar, Pune 411028, Maharashtra, India; dnyanesh.ranade@seruminstitute.com (D.R.); prajwal6902@gmail.com (P.C.); salunkeak1937@gmail.com (A.S.); aniket26.mahamuni@gmail.com (A.M.)

**Keywords:** R21 malaria multi-dose vaccine, virus-like particle (VLP), immunogenicity, 2-phenoxy-ethanol (2-PE), protein estimation, matrix M1, Bradford assay

## Abstract

**Background:** The R21 malaria vaccine is a next-generation, WHO-prequalified vaccine that was introduced to reduce the burden of clinical malaria. In alignment with WHO recommendations, multi-dose vaccine presentations are preferred for large-scale immunization and inclusion in the Expanded Programme on Immunization (EPI). Accurate protein quantification is a critical quality control parameter for lot release, but it remains challenging when the antigen is present at low protein concentrations or formulated with complex matrices, including adjuvants, stabilizers, and preservatives. **Methods:** In this study, multiple protein estimation methods including Micro-BCA, BCA, and Bradford assays were evaluated to determine their suitability for quantifying the R21 antigen formulated with Matrix-M1 adjuvant and 2-PE preservative. The Bradford assay was selected as the most appropriate method, based on a comparative assessment of precision, accuracy, and linearity. Further optimization was undertaken to identify suitable buffer systems, and the method was validated in accordance with ICH Q2(R2) guidelines. **Results:** Validation results demonstrated that the assay is specific, accurate, precise, and repeatable, with a limit of quantitation (LOQ) of 2 µg/mL. The method demonstrated comparable performance to ELISA and was found to be sensitive enough to detect changes in antigen concentration resulting from unintended adsorption of R21 to vial surfaces. The assay offers a rapid, high-throughput, and cost-effective solution for protein quantitation in commercial manufacturing, lot release, and stability studies. The protein content of the drug product, quantified using the Bradford method, demonstrated robust in vivo immunogenicity in both release and stability studies. **Conclusions:** The robustness and reproducibility of the assay establish a new benchmark in quality control for virus-like particle (VLP)-based vaccines with complex formulations, thereby supporting the precision and reliability required for global malaria prevention efforts.

## 1. Introduction

Malaria, primarily caused by *Plasmodium falciparum*, is a life-threatening disease and the leading cause of malaria-related deaths globally [1,2]. *P. falciparum* is known for its severe, rapidly progressing infections [3,4,5], prompting the development of targeted vaccines like RTS,S/AS01 and R21/Matrix M1 [6,7]. R21/Matrix M1, a next-generation vaccine, offers key advantages over RTS,S, including higher scalability, a significantly lower dose requirement (one-fifth of RTS,S), a single-vial formulation (antigen and adjuvant co-formulated), and improved cost-effectiveness, positioning it as a promising tool in the global fight against malaria [8]

In alignment with the WHO’s goal of eliminating malaria in at least 30 countries by 2030 and recognizing the logistical and programmatic advantages of multidose vaccine presentations over single-dose formats, especially for mass immunization in resource-limited settings, a multidose formulation of the R21 malaria vaccine is currently under development [9,10]. One of the primary challenges in such formulations is preventing microbial contamination during repeated dose withdrawals, typically over a period of at least 28 days; as outlined in the WHO’s multidose vaccine policy [11], repeated vial piercing in multidose presentations increases the risk of contamination, necessitating the inclusion of a suitable preservative to ensure in-use stability [12]. To address this, the R21 malaria vaccine is being formulated with 2-PE as a potential preservative [13].

Any change in vaccine formulation, however, necessitates the redevelopment of quality control parameters: most critically, the method for protein concentration estimation. This parameter is vital for ensuring batch-to-batch consistency, detecting container closure integrity issues, and monitoring antigen content to achieve the desired immunopotency [14]. In multidose drug products (DP), protein estimation becomes especially challenging when the antigen is present at very low concentrations (≤20 µg/mL). The complexity is further compounded by the presence of adjuvants such as Alhydrogel, AdjuPhos, and saponin-based systems, along with excipients like potassium, magnesium, ammonia, EDTA, Tris, carbohydrates, and reducing agents. These substances can interfere with conventional protein quantification techniques, thereby compromising the accuracy and reliability of the results [15,16,17,18].

In most vaccines, protein estimation is typically conducted at the bulk or intermediate stages, where antigen concentrations are relatively high, to circumvent the analytical challenges associated with low-dose formulations. However, this approach does not fully address the necessity of protein quantification at the drug product level, where the final formulation may affect antigen stability and potency. While some studies have reported protein quantification following antigen extraction from the final formulation, such methods often suffer from low reproducibility and a high risk of inaccurate results, due to variable recovery during the extraction process [19].

Direct methods such as the O-Phthalaldehyde (OPA) assay and Bis-ANS assay have been employed for protein quantification; however, they have limited applicability in industrial settings [20,21]. Moreover, these methods lack extensive validation data restricting their use for drug product quantification [14]. Given the critical need to determine protein concentration at the drug product stage, a robust and validated assay is essential. Such an assay would support the evaluation of consistency across unit operations, monitoring of process-related losses, control of blending parameters, verification of fill-finish accuracy, and confirmation of product homogeneity and integrity. Additionally, it is crucial for assessing antigen adsorption efficiency to adjuvants, and for conducting stability studies that are necessary for shelf-life determination [22,23].

Initially, an intrinsic fluorescence (IF) method was developed for quantifying low concentrations of Matrix M1 adjuvanted R21 malaria vaccine without preservatives, showing linearity from 4 to 20 µg/mL [14]. However, the addition of 2-PE in the multidose formulation interfered with IF at 280/345 nm, compromising accuracy. In view of these limitations, alternative methods for protein quantifications such as BCA, the Micro-BCA and Bradford methods were explored in the present study to evaluate their feasibility to quantitate the protein concentration of Matrix M1 adjuvanted R21 malaria vaccine with 2-PE.

Given these challenges, the Bradford assay [24] was investigated for its suitability. This study explores its applicability for quantifying the R21 antigen in such formulations. Given that the Bradford method typically uses BSA as a standard, its accuracy may be affected when applied to structurally distinct proteins like recombinant VLP-based antigens. To address this, we conducted a comparative analysis using both BSA and an in-house R21 reference standard, alongside other conventional methods, to evaluate the assay’s reliability and precision.

Based on the outcomes, the Bradford assay was identified as the most suitable method for protein quantitation, following a comparative assessment of precision, accuracy, and linearity. Further optimization was conducted to determine appropriate buffer systems, and the method was validated in accordance with ICH Q2 (R2) guidelines [25]. Validation results confirmed that the assay is specific, accurate, precise, and repeatable, with a limit of quantitation (LOQ) of 2 µg/mL. The method demonstrated performance that was comparable to ELISA and was sufficiently sensitive to detect changes in antigen concentration caused by unintended adsorption of R21 to vial surfaces. Moreover, the protein quantified through the Bradford method, when applied to in vivo potency assays in mice, also resulted in comparable seroconversion relative to IHRS. The robustness and reproducibility of the adapted Bradford assay set a new benchmark for quality control testing of virus-like particle (VLP)-based vaccines with complex formulations, supporting the precision and reliability that is essential for global malaria prevention initiatives.

## 2. Materials and Methods

### 2.1. Reagents and Consumables

The R21 malaria vaccine was developed by Jenner Institute, (Oxford, UK) and has been commercialized by Serum Institute of India Private Limited (SIIPL), Pune, India. Multi-dose presentations of the R21 malaria vaccine containing 2-PE as a preservative were formulated at SIIPL, with the R21 antigen concentration at 13 µg/mL. Bradford reagent, BSA, Tween 20 and 2-PE were procured from Sigma Aldrich (St. Louis, MO, USA). The multimode microplate reader was procured from Molecular Devices (San Jose, CA, USA) and the densitometer for gel analysis was procured from GE HealthCare (Chicago, IL,USA). The monoclonal primary antibody was developed and qualified in-house, while the anti-rabbit IgG, conjugated with HRP, was procured from R&D systems (Minneapolis, MN, USA). Anti-mouse IgG, conjugated with HRP, was procured from R&D systems. UV transparent plates (UVT) for 2-PE estimation were procured from BRAND Scientific (Wertheim, Germany). Matrix M1 adjuvant was obtained from Novavax (Gaithersburg, MD, USA). ELISA plates (Nunc MaxiSorp flat bottom), SDS-PAGE molecular weight markers, and the micro BCA protein assay kit were procured from Thermo Fisher Scientific (Waltham, MA, USA). Pre-cast 4–15% tris-glycine SDS-PAGE gels were obtained from Biorad Laboratories (Hercules, CA, USA), and the silver staining kit was procured from Thermo-Scientific (Waltham, MA, USA). Rabbits used for polyclonal antibody generation and BALB/c mice used for the in vivo potency assay were housed at the SIIPL animal facility, with due approval from IAEC (CPCSEA), IAEC protocol number 04/25-03/2024-MT dated 25.03.2024, 06/30-09/2024-MT dated 30.09.2024, and 06/26-03/2025-MT dated 26.03.2025. Statistical analysis for the applicable data has been conducted using Microsoft Excel.

### 2.2. Characterization Studies for R21+2-PE

#### 2.2.1. SDS-PAGE Followed by Silver Staining

An in-house reference standard (IHRS) of R21 and a representative batch of the R21 multidose formulation containing 2-PE (R21+2-PE) were mixed with reducing sample buffer in an appropriate ratio and heated at 100 °C for 10 min to ensure denaturation. Appropriate volumes of each sample were then loaded onto a 4–15% tris-glycine gradient SDS-PAGE gel. Electrophoresis was performed at a constant voltage of 120 V for 1 h. Following separation, protein bands were visualized using silver staining. The stained gel was imaged and subjected to densitometric analysis to evaluate the presence, integrity, and banding pattern of the antigens, thereby assessing their structural consistency and protein profile.

#### 2.2.2. Western Blotting

For Western blotting, SDS-PAGE was performed as described in the previous section. Following electrophoresis, the separated proteins were transferred onto a nitrocellulose membrane using standard blotting conditions. The membrane was then blocked with 5% BSA solution to prevent non-specific antibody binding, followed by thorough washing using phosphate-buffered saline with Tween-20 (PBST). The membrane was subsequently incubated with a primary antibody that was specific to the R21 antigen. After additional washes to remove unbound primary antibody, the membrane was incubated with a horseradish peroxidase (HRP) conjugated secondary antibody. Antigen specific protein bands were visualized using DAB-urea as the chromogenic substrate, resulting in the development of clearly defined bands corresponding to the R21 antigen.

#### 2.2.3. Antigenic Ratio by In Vitro ELISA

The antigenic ratio of the R21+2-PE malaria vaccine formulation was determined using an in vitro ELISA. An IHRS of R21 and the R21+2-PE malaria vaccine test sample were coated in triplicate onto 96-well Nunc MaxiSorp™ flat-bottom plates and incubated overnight at 4 °C. The IHRS was serially diluted from 1 µg/mL to 0.457 ng/mL, and the test sample was coated at a corresponding concentration.

Following the overnight coating, the plates were blocked with blocking buffer for 2 h at room temperature to prevent non-specific binding and then washed three times with phosphate-buffered saline with Tween-20 (PBST). An anti-R21 polyclonal primary antibody was added at an appropriate dilution and incubated at 37 °C. After incubation, the plate was washed again, and an HRP conjugated anti-rabbit secondary antibody was added, followed by a 1 h incubation at 37 °C.

Next, 100 µL of TMB/H_2_O_2_ substrate solution was added to each well, and the plate was incubated at 37 °C for 15 min in the dark. The enzymatic reaction was stopped by adding 100 µL of 2N H_2_SO_4_. Absorbance was measured at (lm1: 450 nm) and (lm2: 630 nm), using a multimode 96-well plate reader. Antigen concentrations of the test samples were calculated using SoftMax^®^ Pro software (Version 7.0.2), based on a four-parameter logistic (4PL) standard curve. The standard curve was generated using the IHRS of R21+2-PE, and test samples were diluted 27-, 81-, and 243-fold. Optical density (OD) values obtained for the test samples were interpolated against the IHRS curve to determine antigen concentrations. The IHRS was assigned a relative antigenic value of 1, and the antigenic ratio of the R21+2-PE malaria vaccine test samples was determined accordingly. All experiments were performed in triplicate, and the standard deviations were plotted.

#### 2.2.4. Estimation of 2-PE in R21 Multidose Presentation

Quantification of 2-PE in the R21 multidose formulation was performed, following the method described by Ranade et al. (2023) [13]. Briefly, a standard curve of 2-PE was generated in the concentration range of 0.10% to 0.30%, using PBS as the diluent. The standards were prepared on a 96-well UV-transparent plate. Test samples were appropriately diluted to fall within the linear range of the standard curve and added to the plate in parallel with the standards. The absorbance of both the standards and diluted test samples was measured at 245 nm using a multimode plate reader. The concentration of 2-PE in the test samples was determined by interpolating the corresponding absorbance values against the standard curve. Values of reference standard of the R21+2-PE and R21+2-PE samples were also incorporated in the applicable figure. The experiment was performed in triplicate and the standard deviation was plotted.

### 2.3. Protein Estimation of R21+2-PE Formulation by Different Protein Quantification Methods Using BSA and IHRS as Standards

Protein quantification in the R21 multidose formulation containing 2-PE was evaluated using three different methods: Bicinchoninic Acid (BCA), Micro BCA assay, and the Bradford assay. Each method was assessed using both like-to-like in-house reference standards (IHRS) of R21 and BSA standard. The objective of the study was to down-select the most suitable protein estimation method and to evaluate the feasibility of using IHRS or BSA as the calibration standard. In the BCA assay, appropriate volumes of standards and samples were added in duplicate to the wells, followed by the addition of the BCA working reagent. The plate was incubated at 37 °C for 30 min, and absorbance was measured at 562 nm. For the Micro BCA assay, the working reagent was freshly prepared by mixing Micro BCA Reagents A, B, and C in a ratio of 25:24:1. Standards and samples were added to the wells, along with the working reagent, and incubated at 37 °C for 2 h. Absorbance was then measured at 562 nm.

In the Bradford assay, the standards were prepared in a 2-PE-based buffer containing 10 mM Tris, 15 mM MgCl_2_, and 7.5% sucrose, with a protein concentration range of 4–20 µg/mL. Each standard and undiluted test sample (100 µL) was added per well, followed by 100 µL of Bradford reagent, yielding a final volume of 200 µL per well. The plate was incubated at an ambient temperature, protected from light using aluminum foil, and absorbance was measured at 595 nm. Protein concentrations for R21+2-PE samples were calculated using calibration curves generated with both BSA and IHRS standards. Standard deviations for each method were analyzed to assess reproducibility and precision. Based on these results, the Bradford assay using the IHRS as standard was selected for all subsequent protein estimation studies for the R21 multidose formulation.

### 2.4. Optimization of Buffers for Protein Estimation of R21+2-PE Using Bradford Method

Three distinct buffer systems were assessed to determine their influence on protein quantification using the Bradford assay: Buffer A (PBS), Buffer B is the formulation buffer without Matrix M1 adjuvant (formulation buffer containing 7.5% sucrose, 15 mM MgCl_2_, and 10 mM Tris, and 2-PE), and Buffer C is the complete formulation buffer (Buffer B with Matrix M1 adjuvant). Standard calibration curves ranging from 4 to 20 µg/mL were prepared using each buffer system. The R21+2-PE malaria vaccine samples were then quantified against these individual buffers to assess the impact of buffer composition on assay performance. The final volume for each well was adjusted to 100 µL, followed by an addition of 100 µL of Bradford reagent. The plate was incubated at ambient temperature for 30 min and protected from light using aluminum foil. Absorbance was measured at 595 nm using a microplate reader. Protein concentrations of the test samples were calculated using the calibration curve prepared with the IHRS in various buffers.

### 2.5. Method Validation Studies

Method validation was conducted in alignment with the ICH Q2(R2) guidelines for the validation of analytical procedures. The validation focused on key parameters such as specificity, limit of quantification (LOQ), accuracy, intermediate precision, and repeatability. Specificity was assessed by evaluating potential matrix interference from the formulation buffer. A volume of 100 µL of the R21+2-PE multidose formulation (13 µg/mL) and Buffer B, Buffer C, PBS alone, and 2-PE at a concentration of 11 mg/mL prepared in PBS were each added in duplicate to a microtiter plate, followed by the addition of 100 µL of Bradford reagent. The plate was incubated at an ambient temperature for 30 min. Post-incubation, absorbance spectra were recorded across the visible light range of 400–700 nm. Spectral subtraction using Buffer B as baseline was performed to confirm the absence of matrix interference and ensure signal specificity to the protein.

Limit of quantification (LOQ) was established by analyzing a series of dilutions of the R21+2-PE malaria vaccine, ranging from 0.5 to 20 µg/mL. The LOQ was determined based on the lowest measured concentration with acceptable percentage recovery (within 80–120%) and %coefficient of variation (CV) (<20%). Accuracy was evaluated by preparing samples at four different concentrations, 6.5 µg/mL, 9.75 µg/mL, 13 µg/mL, and 16.25 µg/mL, corresponding to 50%, 75%, 100%, and 125% of the target concentration (13 µg/mL). Protein estimation was performed using the Bradford assay and a calibration curve was plotted to compare observed versus theoretical concentrations.

Intermediate precision was determined by analyzing R21+2-PE malaria vaccine samples by two independent analysts on two separate days. The %CV between the two data sets was calculated to assess the variability attributable to analyst and day to day factors. Repeatability was assessed by analyzing six independent batches of the R21+2-PE malaria vaccine multidose formulation, each in triplicate. The intra assay %CV was calculated to evaluate repeatability under the same analytical conditions. All validation experiments were conducted in triplicate (*n* = 3), and standard deviations were plotted for each parameter to visualize variability and assess the robustness and reproducibility of the method.

### 2.6. Method for Estimation of Protein Concentration of R21+2-PE Batches

#### 2.6.1. Comparative Assessment of Protein Concentration of GMP Batches of R21+2-PE Malaria Vaccine by Bradford and ELISA Method

The validated Bradford assay was subsequently employed to quantify the protein concentration of GMP batches of the R21+2-PE multidose formulation, as described earlier. Following protein quantification, an antigenic content estimation was performed using an in vitro ELISA, in accordance with the method reported by Ranade et al. [14] In this ELISA, the antigenic content of each GMP trial batch was calculated using the following formula:

Protein content by ELISA (µg/mL) = Protein concentration obtained by Bradford method × Antigenic ratio

The protein concentrations determined by the Bradford assay were then compared with those derived from the ELISA-based antigenic estimation, to understand the correlation between both the assays. All analysis was performed in triplicate (n = 3), and standard deviations were plotted to evaluate batchwise variability and assay consistency.

#### 2.6.2. Differentiation of Siliconized vs. Improperly Siliconized Vials Through Protein Estimation of R21+2-PE Batches

The Bradford assay was employed to differentiate between properly siliconized and improperly siliconized glass vials by estimating the protein concentration of R21+2-PE batches. Improperly siliconized vials were identified based on the presence of a surface meniscus in the filled vials, which is indicative of uneven or inadequate siliconization. Protein estimation was conducted for vaccine batches filled into both properly and improperly siliconized vials. Each condition was tested in triplicate (n = 3), and the results were presented with standard error bars to assess variability and reproducibility.

#### 2.6.3. In Vivo Potency Assay to Estimate Immunogenicity of R21+2-PE Batches

The in vivo potency assay for R21+2-PE was conducted in BALB/c mice. Groups of ten mice, aged six to eight weeks, received a single intraperitoneal immunization with a series of five graded dilutions of both the test vaccine and the in-house reference standard. Terminal bleeds were collected four weeks post-immunization, and serum samples were analyzed by ELISA to quantify anti-R21 antibody responses. Seropositivity for each dilution group was determined using a cutoff value established from buffer control samples. Relative potency was calculated using probit analysis in CombiStats software (Version 1.2.1), comparing the test vaccine to the IHRS.

Potency testing was performed on clinical lots of R21+2-PE during lot release and throughout stability monitoring. Stability evaluations included real-time storage at 2–8 °C for up to 12 months, as well as stress studies at 40 °C for four weeks.

In addition, in-use stability potency studies were performed on three batches to simulate multi-dose administration conditions. For this study, a dose volume of 0.5 mL was withdrawn from the same 10 dose vial, using 22–25 gauge needles for nine sequential doses. The repeated needle insertions were intended to mimic routine clinical practice and potential perturbation of the container closure. The vial was stored at 2–8 °C between withdrawals, with doses removed at three-day intervals over 27 days. Following the ninth withdrawal, the remaining tenth dose in each vial was subjected to in vivo potency testing.

## 3. Results

### 3.1. Characterization Results for R21+2-PE

**Figure 1:**
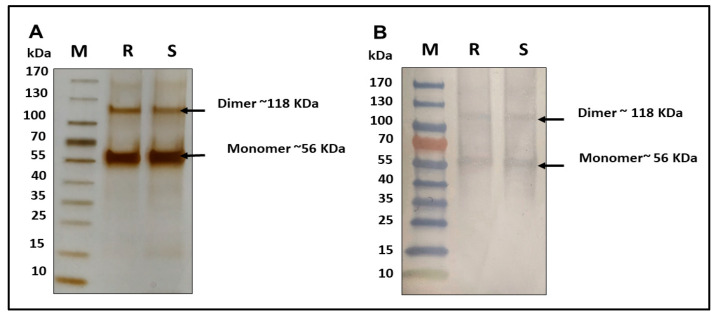
Characterization of R21+2-PE malaria vaccine. (**A**): SDS-PAGE followed by silver staining, (**B**): Western blotting using anti-R21 polyclonal antibody. (M: marker, R: reference standard of R21+2-PE, and S: sample of R21+2-PE).

The R21+2-PE malaria vaccine underwent comprehensive characterization encompassing purity profiling using SDS-PAGE, identity confirmation using Western blotting, antigenicity assessment using ELISA, and 2-PE content estimation. Purity analysis via reducing SDS-PAGE coupled with silver staining (Figure 1A) revealed the presence of a prominent monomer of R21 protein, which appeared in the region of approximately 56 kDa and a dimer at 118 kDa. Notably, this electrophoretic profile of R21 bands demonstrated congruence with the established IHRS. Apart from the monomer and dimer of R21, no other band was noticed in the gel, which ascertains the high degree of purity of antigen (detailed analysis of densitometry appended in the Supplementary Information). Furthermore, identity was affirmed through Western blotting (Figure 1B), wherein both the monomeric and dimeric forms of the protein exhibited positive immunoreactivity with anti-R21 polyclonal antibodies, which were raised in-house.

**Figure 2:**
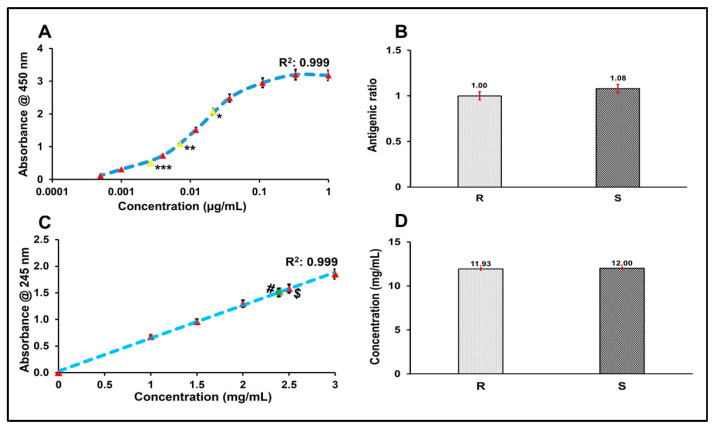
Characterization of R21+2-PE malaria vaccine. (**A**): Concentrations considered for standard curve are denoted by red colour marker while sample dilutions of * 27, ** 81, and *** 243 fold were represented by yellow marker in standard curve, (**B**): antigenic ratio of R21+2-PE malaria vaccine sample, (**C**): standard curve of 2-PE and values of R denoted by (#) and S denoted by ($), (**D**): 2-PE content estimation of R and S, R: reference standard of R21+2-PE malaria vaccine, and S: sample of R21+2-PE malaria vaccine.

Assessment of the antigenic potency of the vaccine via an indirect enzyme-linked immunosorbent assay (ELISA) (Figure 2A,B) indicated robust antigenicity, with the antigenic ratio relative to the IHRS determined to be approximately 1.08, suggesting comparable immunogenic potential. Quantification of the preservative 2-PE using UV (245 nm) of the R21+2-PE formulation revealed a concentration of 12 mg/mL, which aligns with the theoretical concentration of 2-PE, which was added during the formulation of the fill finish product, precisely with the acceptable limits for vaccine formulations (Figure 2C,D).

### 3.2. Protein Estimation of R21+2-PE Malaria Vaccine by Different Protein Quantification Methods Using Standard Curves of BSA and IHRS

**Figure 3:**
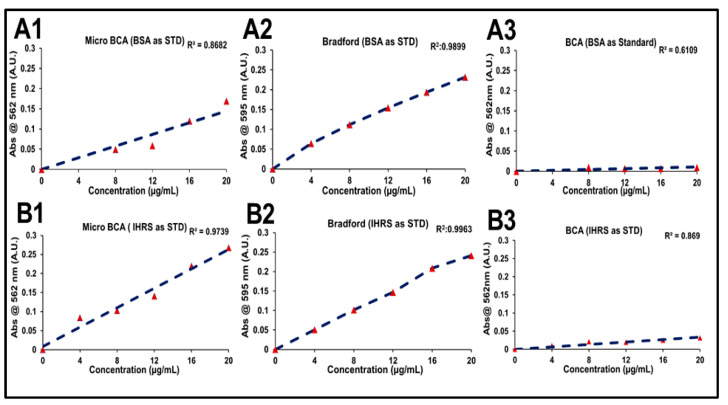
Standard curves used in protein quantification of R21+2-PE malaria vaccine samples using BSA and IHRS. (**A1**) Micro-BCA with BSA, (**B1**) Micro-BCA with IHRS, (**A2**) Bradford with BSA, (**B2**) Bradford with IHRS, (**A3**) BCA with BSA, and (**B3**) BCA with IHRS.

**Figure 4:**
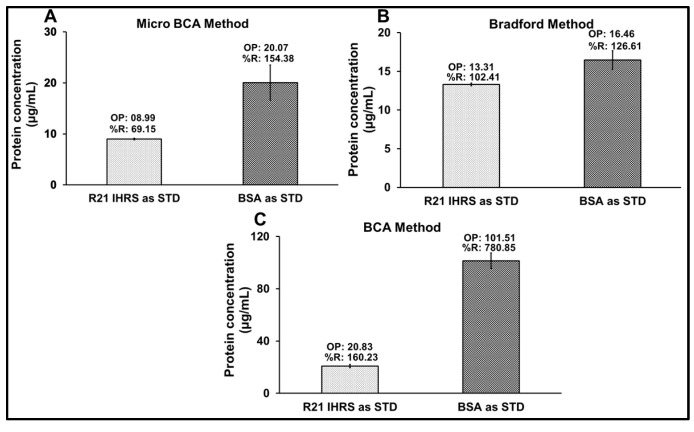
Protein quantification of R21+2-PE by different methods, using standard calibration curve of BSA and IHRS. (**A**) Micro BCA method, (**B**) Bradford method, and (**C**) BCA method. (OP: Observed protein concentration, and %R: % recovery).

Protein quantification of the R21+2-PE multidose formulation was conducted using three analytical methods: Micro-BCA, Bradford assay, and BCA assay. Each method was assessed using two types of standards: IHRS and BSA. Across all quantification approaches, calibration curves generated with IHRS consistently demonstrated superior linearity and accuracy compared to those prepared with BSA (Figure 3). Moreover, protein concentration results of three analytical methods have been presented in Figure 4.

However, among the methods utilizing IHRS, the BCA assay showed the lowest R^2^ value compared to the other techniques. In the Micro-BCA assay, IHRS showed better linearity (R^2^ = 0.9739) than BSA (R^2^ = 0.8682), with relatively better protein estimation (8.99 µg/mL, 69.15% recovery vs. 20.07 µg/mL, 154.38%). The Bradford assay showed strong linearity for both standards (R^2^ > 0.98), but IHRS again provided better accuracy (13.31 µg/mL, 102.41% vs. 16.46 µg/mL, 126.62%). The BCA assay showed poor linearity (R^2^ = 0.8690 for IHRS; 0.6109 for BSA) and significant overestimation, particularly with IHRS (101.51 µg/mL; 780.85%). Overall, IHRS improved assay accuracy, with the Bradford method showing the most reliable performance.

These results, as reflected in the corresponding figures, demonstrate that using IHRS as the calibration standard improves linearity and accuracy across all assays. Among the methods evaluated, the Bradford assay using IHRS provided the most reliable and consistent protein quantification in the complex R21+2-PE formulation.

### 3.3. Optimization of Buffers for Protein Estimation of R21+2-PE Malaria Vaccine Using the Bradford Method

**Figure 5:**
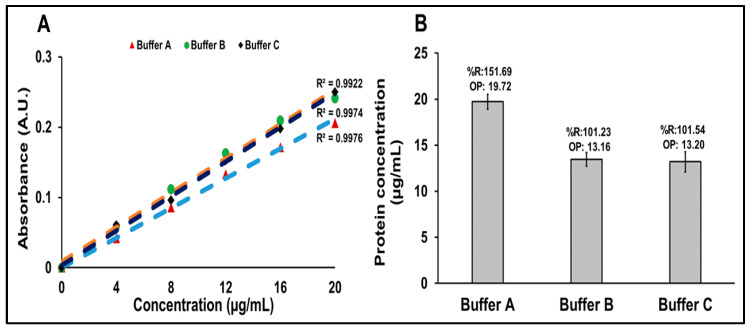
Protein estimation of R21+2-PE malaria vaccine using Bradford method. (**A**) Overlay of standard curves generated for the Bradford protein estimation method with different buffer systems. (**B**) Comparative representation of protein concentrations estimated using the Bradford method, with IHRS curve with different buffer systems as diluents. OP: Observed protein concentration, %R: % recovery.

Three buffer systems, Buffer A, Buffer B, and Buffer C, were evaluated for their impact on R21+2-PE malaria vaccine protein quantification using the Bradford assay. All buffers generated standard calibration curves with strong linearity, as indicated by regression coefficients (R^2^) greater than 0.99. Protein concentrations were then measured using each buffer as a diluent, and percentage recoveries were calculated to assess assay accuracy (Figure 5).

Buffer A (PBS) showed a protein concentration of 19.72 µg/mL (151.69% recovery), exceeding the acceptable range and indicating matrix interference. In contrast, Buffer B and Buffer C yielded recoveries of 101.23% and 101.54%, respectively, within acceptable limits.

These findings indicate that while all buffers supported good calibration curve linearity, Buffer B and Buffer C are more suitable for accurate and reliable protein quantification using the Bradford assay in this formulation.

### 3.4. Validation Studies

**Figure 6:**
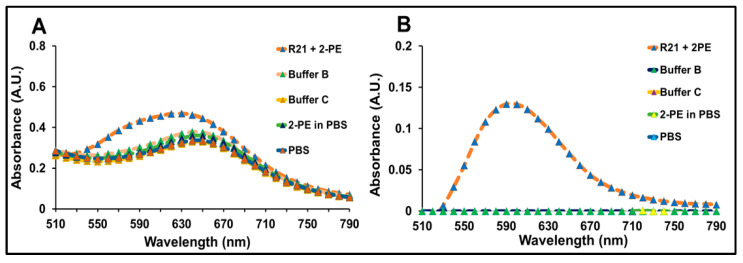
Specificity of Bradford assay: (**A**) visible light range absorption spectra of R21+2-PE, Buffer B, Buffer C, PBS, and 2-PE in PBS, post-addition of Bradford reagent without blank subtraction (**B**) Blank subtracted spectra of R21+2-PE and buffer post-addition of Bradford reagent.

To evaluate the specificity of the Bradford assay, R21+2-PE malaria vaccine sample, Buffer B, Buffer C, 2-PE at concentration of 11 mg/mL constituted in PBS, and PBS buffer alone were analyzed across the visible light range (510 nm–790 nm) following the addition of Bradford reagent and incubation for 30 min, as described in the Materials and Methods Section. Figure 6A presents the uncorrected absorbance, where the R21+2-PE malaria vaccine sample exhibited a characteristic absorbance between 590 and 670 nm, Buffer B, Buffer C, 2 -PE in PBS, and PBS buffer showed a background baseline in the presence of the Bradford reagent. Upon blank subtraction using buffer B (Figure 6B), the R21 +2-PE malaria vaccine spectrum revealed a distinct absorbance maximum at approximately 595 nm, which corresponds to the characteristic absorption of the Bradford dye–protein complex. These results confirm that the assay specifically detects protein in the presence of potential buffer matrix interference through signature spectral shift, thereby affirming its specificity.

**Figure 7:**
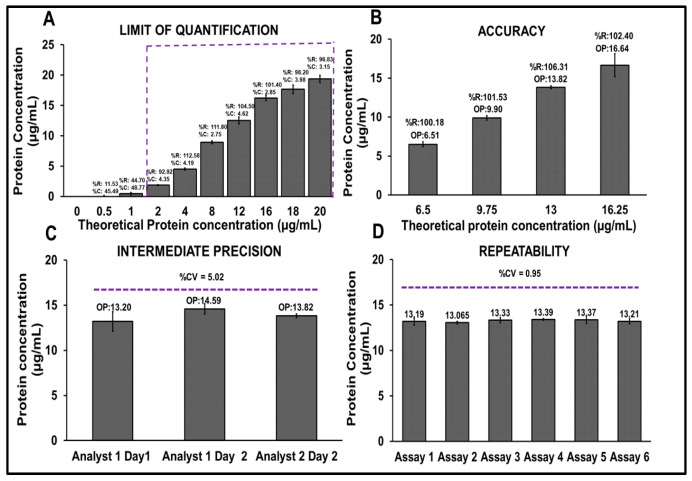
Qualification studies of the Bradford protein estimation method. (**A**) Limit of quantification (LOQ), (**B**) accuracy, (**C**) intermediate precision, (**D**) repeatability. OP: Observed protein concentration, %R: % recovery.

Further method qualification included assessment of the limit of quantification (LOQ), accuracy, repeatability, and intermediate precision (Figure 7A–D). The assay demonstrated a LOQ of 2 µg/mL with acceptable levels of precision and accuracy (Figure 7A). To assess accuracy, four concentrations of R21+2-PE were tested: 6.5 (50%), 9.75 (75%), 13.0 (100%), and 16.25 µg/mL (125%). The percentage recoveries ranged from 100.18% to 106.31%, indicating high assay accuracy (Figure 7B). Intermediate precision evaluated across analysts and days resulted in a %CV of 5.02%, with observed concentrations ranging between 13.20 and 14.59 µg/mL (Figure 7C). Repeatability, assessed through six independent assays at 13 µg/mL, demonstrated excellent reproducibility, with a %CV of 0.95%, (Figure 7D) thereby confirming the robustness of the method.

### 3.5. Results for Estimation of Protein Concentration of R21+2-PE Batches

**Figure 8:**
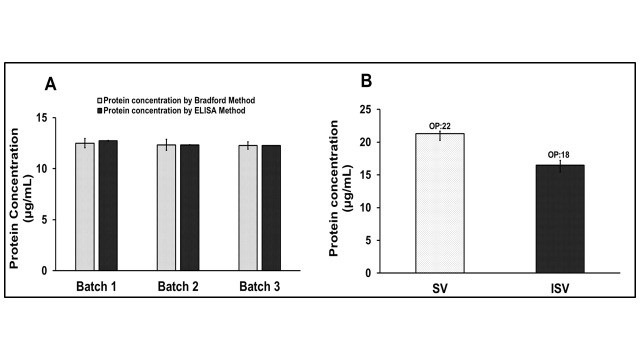
Qualification studies of the Bradford protein estimation method. (**A**) Protein estimation of three GMP batches of R21+2-PE using the Bradford method and ELISA-based method, (**B**) protein concentration of R21 formulation, formulated in siliconized and non-siliconized vials. OP: Observed protein concentration, SV: siliconized vials, and ISV: improper siliconized vials.

Three GMP batches of R21+2-PE were analyzed for their protein content, using both the Bradford assay and an ELISA-based method (Figure 8A). Results from both assays demonstrated comparable protein concentrations across all three batches, with values consistently being within 13 µg/mL ± 1%. This high level of agreement confirms the reliability, accuracy, and concurrence of the Bradford assay with the established ELISA method.

Additionally, the Bradford assay was employed to assess the impact of vial surface properties on protein recovery (measured concentration) for the R21 malaria vaccine, which is a single vial variant, without the adjuvant, formulated at no less than 20 µg/mL. The vaccine was filled into both properly siliconized and improperly siliconized vials and the protein concentrations were evaluated (Figure 8B). Protein concentration, estimated using the Bradford method, was observed at 22 µg/mL in properly siliconized vials, compared to the original fill concentration of 26 µg/mL. In contrast, improperly siliconized vials exhibited a significantly lower measured concentration of 18 µg/mL, likely due to increased protein adsorption to the vial surface.

### 3.6. Animal Assays to Evaluate Immunogenicity of R21+2-PE Batches

In vivo potency assays were performed in BALB/c mice to determine the relative potency of R21+2-PE vaccine batches intended for clinical evaluation. The assay was based on the comparative immunogenicity of the test sample against the IHRS, measured through ELISA. Appropriate dose dilutions of the vaccine were selected to generate measurable antibody responses, such that when the sera were analyzed by ELISA using R21 antigen-coated plates, a gradient of seroconversion was obtained based on a predefined cut-off value. The proportion of seroconverted animals across dose groups was subjected to probit analysis in CombiStats software (Version 1.2.1) to derive the relative potency estimate.

**Figure 9:**
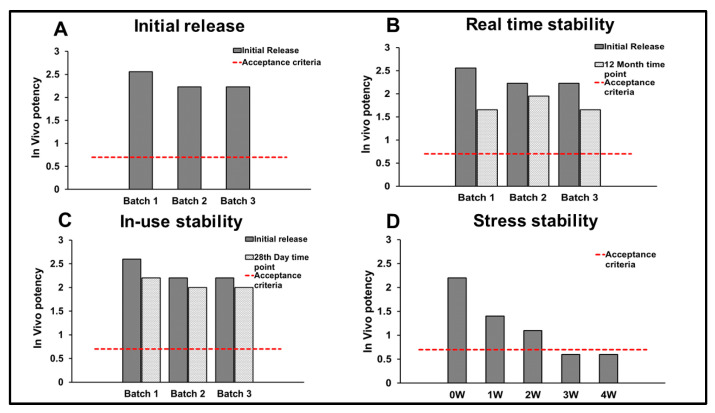
In vivo potency assays for clinical batches of R21+2-PE. (**A**) Upper confidence limits (UCL) for the relative potency (in vivo) of (3) clinical batches of R21+2-PE; (**B**) UCL for the relative potency (in vivo) of (3) clinical batches of R21+2-PE for release and stability at 12 month time point, (**C**) UCL for the relative potency (in vivo) of (3) clinical batches of R21+2-PE for in-use stability and (**D**) UCL of the relative potency (in vivo) of R21+2-PE clinical batch, post-exposure to the stress condition at 40 °C for (1–4 weeks). Acceptable limit (Acc. Limit) for in vivo potency: 0.7 with respect to upper confidence limits of relative potency.

Three clinical batches of R21+2-PE were evaluated, and all batches demonstrated upper confidence limits (UCL) for the relative potency greater than two, indicating equivalence to the IHRS and compliance with the acceptance criterion (UCL ≥ 0.7) (Figure 9A). Additionally, these batches were placed in real-time stability at 2–8 °C for 12 months. Relative potency values obtained post-storage remained within approved specifications, confirming the stability of the vaccine under the recommended storage conditions (Figure 9B).

In accordance with WHO recommendations for multidose presentations, in-use stability was assessed by repeated vial puncturing to withdraw ten doses over a 28-day period. Relative potency values determined at the final withdrawal remained within the specification, confirming suitability for multidose use (Figure 9C).

Furthermore, one clinical batch was subjected to stress at 40 °C for 4 weeks to evaluate the impact of temperature excursion on immunogenicity. The vaccine retained potency within specification for up to 2 weeks under stress conditions, after which a decline in relative potency was observed (Figure 9D).

## 4. Discussion

To quantify the R21 antigen in its adjuvanted form, an intrinsic fluorescence (IF)-based method was initially developed due to its high-throughput potential. However, for large-scale manufacturing and alignment with WHO’s programmatic suitability requirements, a multi-dose formulation was pursued. As per WHO’s Multi-Dose Vial Policy (MDVP, 2014), inclusion of an effective preservative is essential to prevent microbial contamination. Accordingly, 2-PE was introduced into the R21 formulation. Preliminary experiments revealed that 2-PE interfered with the IF-based method due to spectral overlap in excitation and emission wavelengths, rendering the approach unsuitable for protein quantification. To overcome this limitation, several protein quantification methods, including Micro-BCA, Bradford, and BCA assays were evaluated at the target protein concentration (~13 µg/mL) in the presence of Matrix M1 and 2-PE. Alternative methods, including BCA and Micro-BCA, were evaluated but proved ineffective due to the low R21 protein concentration and interference from Matrix M1, 2-PE, and sucrose, particularly when using BSA as the standard.

BSA was initially used as a standard; however, recoveries using BCA and Micro-BCA exceeded 150%, indicating substantial overestimation. This was attributed to structural differences between BSA (a globular protein) and R21 (a virus-like particle-based antigen). BCA-based methods were further impacted by excipients like sucrose and 2-PE. These limitations highlighted the need for a more suitable and reliable method for protein quantification in complex vaccine formulations containing such excipients. Given these challenges, the Bradford assay [24] was investigated for its suitability. This study explores its applicability for quantifying the R21 antigen in such formulations. Given that the Bradford method typically uses BSA as a standard, its accuracy may be affected when applied to structurally distinct proteins like recombinant VLP-based antigens. To address this, we conducted a comparative analysis using both BSA and an in-house R21 reference standard, alongside other conventional methods, to evaluate the assay’s reliability and precision. The discrepancy between BSA and R21 recoveries stemmed from protein-specific differences in amino acid composition (e.g., arginine, lysine, histidine), conformation, and interaction with the acidic Bradford reagent. To resolve this, the R21 protein was first quantified at higher concentration, using the BCA assay with BSA, then used as an IHRS for subsequent Bradford-based quantification, ensuring improved accuracy and reproducibility. This strategy aligns with Bradford’s original recommendation and British Pharmacopoeia guidelines, which advocate for using like-to-like standards for protein assays.

Prior to use, the IHRS was characterized by SDS-PAGE and ELISA. SDS-PAGE confirmed the presence of the R21 monomer and dimer forms, which showed positive reactivity with in-house anti-R21 polyclonal antibodies. ELISA further validated the identity and comparability of the antigen in 2-PE-containing formulations. Additionally, 2-PE content was verified to be within ±5% of the target concentration.

Various dilution buffers were also evaluated for the Bradford assay. The primary objective of evaluating various dilution buffers was to assess their compatibility with the Bradford protein estimation assay, particularly in the context of the R21 malaria vaccine’s multidose formulation. Unlike the RTS, S vaccine, R21 is presented in a single-vial format containing the antigen, adjuvant (Matrix M1), and preservative (2-PE). This complex formulation poses a challenge for accurate protein quantification, due to potential matrix interferences.

Matrix M1 itself is a combination of two saponins, Matrix A and Matrix C, mixed in a defined ratio and at low volumes. The preparation of this formulation buffer is intricate and may introduce variability or operational errors when prepared in smaller quantities. To mitigate this risk, alternative buffer systems were evaluated to identify a diluent that ensures assay accuracy while simplifying operational requirements.

The R21 antigen is formulated in a sucrose-based buffer containing 7.5% sucrose, 10 mM Tris, 15 mM MgCl_2_, Matrix M1, and 2-PE. Hence, a suitable diluent should ideally mimic this composition while minimizing assay interference. Among the tested buffers, PBS (Buffer A) resulted in protein recovery values with more than 20% variability, rendering it unsuitable for accurate protein quantification. In contrast, both Buffer B and Buffer C produced results within the acceptable recovery range (±20%), indicating their compatibility with the assay. The presence of Matrix M1 did not adversely affect the Bradford assay’s performance, demonstrating no significant interference. A background baseline was observed with all buffers when mixed with the Bradford reagent, necessitating its subtraction to accurately determine protein concentration. This background absorbance was consistent and independent of the buffer composition. Protein quantification using buffers such as formulation buffer without Matrix M1, i.e., Buffer B, and complete formulation buffer, i.e., Buffer C, demonstrated high accuracy, indicating that either buffer is suitable for reliable protein estimation.

However, due to the practical challenges associated with the preparation of Matrix M1, especially the requirement to mix Matrix A and Matrix C in precise ratios at low volumes, it was deemed operationally efficient to use the formulation buffer without Matrix M1 (Buffer B) for the Bradford protein estimation. This approach ensures both analytical reliability and ease of buffer preparation.

The Bradford method was validated per ICH Q2(R2) [25] and FDA guidelines. For the evaluation of specificity, the spectrum was obtained with and without blank subtraction. Blank subtraction was performed using the formulation buffer without Matrix M1. This blank subtraction was performed for all further experiments. The assay demonstrated excellent repeatability with a %CV of <0.95% (n = 6) and intermediate precision with a %CV of 5.02% across analysts and days. Accuracy ranged from 80 to 120% recovery (measured concentration), with <5% CV across concentrations. The LOQ was determined to be 2 µg/mL, confirming the method’s sensitivity for use in commercial applications.

Protein estimation is a critical quality attribute in vaccine manufacturing for batch release, monitoring process consistency, and assessing formulation stability. The validated Bradford method was successfully applied to GMP batches of R21+2-PE, yielding protein concentrations comparable to ELISA. Moreover, the method proved sensitive in detecting protein loss due to vial-surface interactions. In container closure studies, the assay differentiated between properly and improperly siliconized vials, identifying lower recoveries in non-siliconized vials, which were likely due to adsorption. This reinforces the need for siliconized containers during routine filling, particularly when sucrose is used as a stabilizer.

In vivo potency is a critical component of vaccine characterization and is directly influenced by the accuracy of protein content determination, as the animal dosing regimen is based on the protein concentration. Therefore, precise protein quantification is essential to ensure appropriate immunization, seroconversion, and reliable estimation of relative potency.

Relative potency assays were performed to evaluate the immunogenicity of the clinical batches of R21+2-PE in BALB/c mice. All three batches demonstrated robust immunogenicity, as evidenced by seroconversion profiles, and were comparable to the IHRS. During long-term real-time stability storage at 2–8 °C for up to 12 months, a slight reduction in relative potency was observed; however, the potency values remained within the established acceptance criteria. This trend confirmed both the stability profile of the vaccine and the sensitivity of the in vivo potency assay in detecting changes in immunogenicity over time.

As R21+2-PE is presented as a multidose formulation, the potential impact of repeated vial puncturing on product integrity was also assessed. In accordance with WHO MDVP recommendations [11], an in-use stability study was conducted to simulate clinical handling, wherein doses were withdrawn over a 28-day period following vial opening. The vaccine retained immunogenicity after the ninth withdrawal, and the remaining tenth dose continued to meet potency specifications, demonstrating suitability for use throughout the recommended in-use period. Furthermore, exposure of the multidose vaccine to accelerated stress conditions at 40 °C showed that the product retained acceptable potency for up to two weeks, indicating resilience to short-term temperature excursions.

Overall, the in vivo potency evaluations confirmed that the R21+2-PE clinical batches, dosed based on protein content determined via the Bradford assay, were immunogenic and maintained potency during real-time storage, in-use handling, and short-term thermal stress conditions.

In conclusion, the Bradford assay was successfully optimized and validated for the quantification of the R21 antigen in complex, adjuvanted multi-dose formulations containing 2-PE. This method addresses key challenges including low antigen concentration, excipient interference, and the importance of a like-to-like reference standard. It offers a reliable, high-throughput, and cost-effective solution that is suitable for routine batch release, stability testing, and large-scale manufacturing of the R21 malaria vaccine.

## 5. Conclusions

This study successfully adapted and validated the Bradford method for protein quantification in multi-dose R21 malaria vaccine formulations containing 2-PE and Matrix M1 adjuvant. Unlike conventional approaches, the optimized method addresses the challenges posed by low antigen concentration, presence of 2-PE, excipient interference, and the necessity for a like-to-like reference standard. The validated method provides a rapid, high-throughput, and cost-effective solution for accurate protein quantitation in commercial-scale production, initial batch release, and stability studies. Owing to its robustness and reproducibility, it sets a new quality control standard for VLP-based vaccines formulated with complex excipients, supporting accuracy in commercial vaccine release for worldwide malaria prevention. Moreover, the clinical batches of R21 formulated with 2-PE demonstrated consistent immunogenicity at initial release, after 12 months of real-time stability storage, throughout the 28-day in-use stability period, and for up to two weeks under stress conditions. Clinical evaluation of the multidose formulation is currently ongoing in Mali under the IMVACS trial [26], which aims to compare the safety, reactogenicity, and humoral immunogenicity of the 10-dose (multidose) R21/Matrix-M formulation containing 2-Phenoxyethanol (2-PE) with that of the standard 2-dose R21/Matrix-M formulation.

## Data Availability

The authors confirm that the data supporting the findings of this study are available within the article.

## Supplementary Materials

The following supporting information can be downloaded at: https://www.mdpi.com/article/10.3390/vaccines14010025/s1. Analysis Report: Malaria Vaccine (Recombinant, Adjuvanted) with 2-PE.

## Abbreviations

The following abbreviations are used in this manuscript:

WHO	World Health Organization
EPI	Expanded Programme on Immunization
2-PE	2-Phenoxy Ethanol
LOQ	Limit of Quantification
VLP	Virus Like Particles
EDTA	Ethylenediamine Tetra Acetic Acid
ELISA	Enzyme-Linked Immunosorbent Assay
ANS	Aniline Sulfonic Acid
BCA	Bicinchoninic Acid Assay
UV	Ultraviolet
BSA	Bovine Serum Albumin
ICH	International Council for Harmonisation of Technical Requirements for Pharmaceuticals for Human Use
IHRS	In-House Reference Standard
USP	United States Pharmacopeia
GMP	Good Manufacturing Practices
SIIPL	Serum Institute of India Private Limited
PBS	Phosphate-Buffered Saline
IF	Intrinsic Fluorescence
PBST	Phosphate-Buffered Saline with Tween-20
UCL	Upper Confidence Limits
IAEC	Institutional Animal Ethics Committee
CPCSEA	Committee for the Purpose of Control and Supervision of Experiments on Animals
IMVACS	Integrating Malaria Vaccine with Seasonal Malaria Chemoprevention in West Africa
