# Supplementary material for: Systematic Development and Validation of a Bradford-Based Protein Quantification Method for Novel Multi-Dose R21 Malaria Vaccine Formulated with 2-Phenoxy Ethanol (2-PE)
Source: Vaccines (Basel). 2025 Dec 24;14(1):25. doi: 10.3390/vaccines14010025 (PMC12846647; doi:10.3390/vaccines14010025)
Supplement: Supplementary file 1 [file vaccines-14-00025-s001.zip › vaccines-4020843-supplementary.pdf]

Analysis Report: Malaria Vaccine (Recombinant, Adjuvanted) with 2-PE

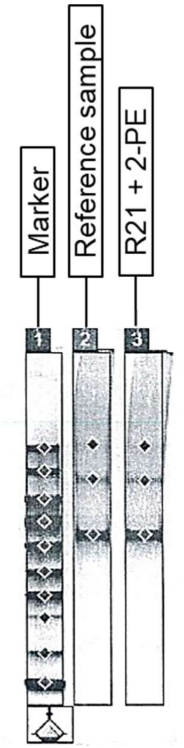

Lane 1: Marker

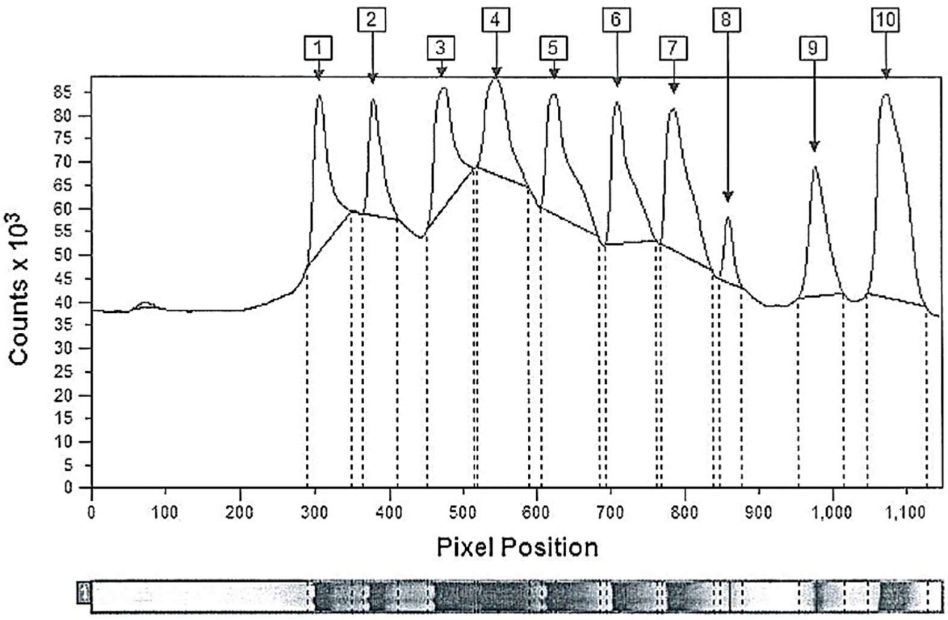

Lane 2: Reference Sample

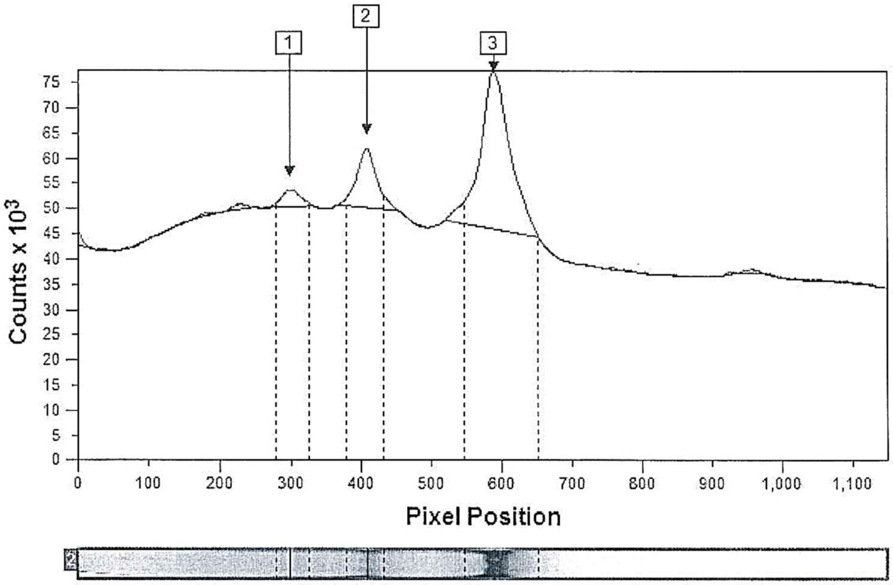

Band Table

Band No	Volume	Area	Band %	MW (kd)	Rf
1	12267554.97	5400	5.201	174.988	0.260
2	42742263.06	6240	18.122	119.455	0.356
3	180844110.85	12600	76.676	63.465	0.514

Lane 3: R21 + 2-PE

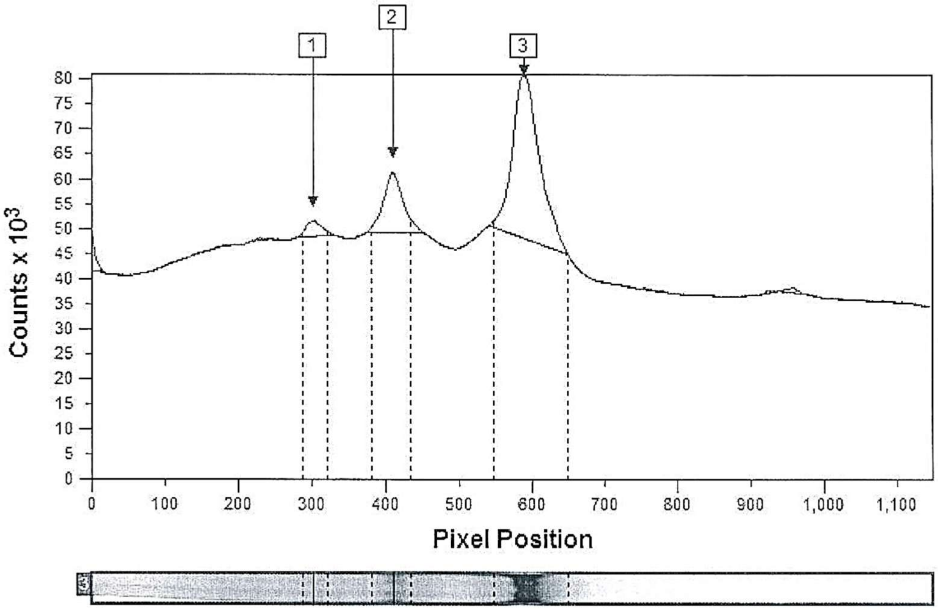

Band Table

Band No	Volume	Area	Band %	MW (kd)	Rf
1	8920880.40	4130	4.042	171.982	0.264
2	41523680.06	6136	18.816	118.627	0.358
3	170238775.27	12036	77.142	63.465	0.514
